# 

**Published:** 1898-10-01

**Authors:** 


					CADBURY'S ^ o CADBURY'S
/ COCOA
COCOA
ABSOLUTELY PURE. ^ NO DRUGS OR ALKALIES USED.
I
No. 627 Vol. XXV. [SfiSSJSO Saturday,
Ocr. 1st, 1898.
~MUftf.ULn.Aua MUfilY/CH HUSifAJAU
[SubeeruptionJTcrm8?] pRICE TWOPENCE.

				

